# Disease-Suppressive Activity of Lecithin Against Foliar Infection by *Rhizoctonia solani* Isolates in Cabbage, Rice, and *Brachypodium distachyon*

**DOI:** 10.3390/life16060998

**Published:** 2026-06-13

**Authors:** Tran Xuan Cuong, Misaki Asano, Daiki Honma, Moeko Soeda, Megumi Watanabe, Nanami Sakata, Hidenori Matsui, Kazuhiro Toyoda, Yuki Ichinose, Kentaro Ikeda, Yoshiteru Noutoshi

**Affiliations:** 1 Graduate School of Environmental, Life, Natural Science and Technology, Okayama University, Okayama 700-8530, Japan; tranxuancuong@hdu.edu.vn (T.X.C.); po7l1am9@okayama-u.ac.jp (M.W.); nanami.sakata@okayama-u.ac.jp (N.S.); hmatsui@okayama-u.ac.jp (H.M.); pisatin@okayama-u.ac.jp (K.T.); yuki@okayama-u.ac.jp (Y.I.); 2 Department of Crop Science, Faculty of Agriculture, Forestry and Fishery, Hong Duc University, Thanh Hoa City 440000, Vietnam; 3 School of Agriculture, Okayama University, Okayama 700-8530, Japan; pd548ojh@s.okayama-u.ac.jp (M.A.);; 4 Faculty of Bioscience and Applied Chemistry, Hosei University, Tokyo 184-8584, Japan; ikeda-ken@hosei.ac.jp

**Keywords:** *Rhizoctonia solani*, foliar symptoms, leaf infection, cabbage, rice, *Brachypodium distachyon*

## Abstract

*Rhizoctonia solani* is a necrotrophic phytopathogenic fungus that causes disease in various crops. In agriculture, many crops suffer from root or seedling rot caused by this soil-borne pathogen, whereas cabbage and rice develop lesion-like symptoms on aboveground tissues. Diseases caused by *R. solani* are generally controlled using chemical fungicides; however, environmentally friendly alternatives are needed for sustainable agriculture. In this study, we evaluated the efficacy of lecithin, a mixture of phospholipids previously registered in Japan as an agrochemical for controlling cucumber powdery mildew, against *Rhizoctonia* diseases. In cabbage, foliar spraying of 0.2–1.0% soybean lecithin effectively suppressed leaf symptoms caused by *R. solani* isolate RhiCa-2, which was identified as AG-1 IB. In rice and *Brachypodium distachyon*, 0.2–1.0% lecithin significantly suppressed leaf symptoms induced by *R. solani* AG-1 IA. Hyphal staining of inoculated leaves revealed reduced hyphal density on lecithin-treated leaves. Consistently, hyphal growth of *R. solani* on cellophane placed on water agar was retarded by lecithin treatment. However, 5.0% lecithin induced phytotoxicity in *B. distachyon*. Egg yolk-derived lecithin also exhibited disease-suppressive activity in cabbage and *B. distachyon*, with efficacy comparable to that of soybean lecithin under the conditions tested. These results suggest that lecithin suppresses foliar infection by *R. solani*, at least in part, through direct inhibitory effects on fungal hyphae, and may serve as a potential alternative material for disease control in sustainable crop production.

## 1. Introduction

*Rhizoctonia solani* is a soil-borne necrotrophic fungus with a broad host range that belongs to the phylum Basidiomycota [1,2]. *R. solani* is a species complex traditionally classified into 13 anastomosis groups (AGs) based on compatibility in hyphal fusion reactions [2,3]. Because certain AGs are frequently isolated from particular *Rhizoctonia* diseases in specific crops, AG classification has long been considered to be associated with host preference or host specificity. However, accumulating evidence indicates that the relationship between AGs and host range is not always straightforward [4,5,6,7,8,9].

Most plants suffer from root or seedling rot caused by *R. solani*, particularly when young seedlings are transplanted into infested soil under disease-conducive conditions. However, some plants develop lesion-like symptoms on aboveground tissues. In rice grown in paddy fields, sheath blight is caused mainly by *R. solani* AG-1 IA. Floating sclerotia attach to rice sheaths and subsequently develop into round lesions, after which aerial hyphae spread to the upper parts of the plant [10,11]. *R. solani* AG-1 IA also causes severe leaf symptoms in detached leaves or plantlets of the model monocotyledonous plant *Brachypodium distachyon* [12,13]. We have previously compiled basic information on *B. distachyon*, including plant hormone-responsive marker genes and the transcriptome associated with pattern-triggered immunity, and have characterized the pathogenicity mechanisms of this fungus by analyzing the responses of this model plant during its interaction with *R. solani* [9,14,15,16]. In addition to monocotyledonous plants, dicotyledonous crops are also severely impacted. For instance, cabbage is affected by several *Rhizoctonia* diseases, including damping-off, wirestem, root rot, bottom rot, and head rot [17,18].

In Japan, fungicides from several chemical classes are used to manage *Rhizoctonia* diseases in rice and cabbage. Under ordinary disease pressure, rice sheath blight may be partially suppressed as a secondary effect of strobilurin-type quinone outside inhibitor (QoI) fungicides, which are primarily applied to control rice blast caused by *Pyricularia oryzae*. However, QoI-resistant isolates of *R. solani* AG-1 IA causing sheath blight have been reported in the United States [19,20]. When sheath blight reaches epidemic levels, fungicides specifically targeting this disease are used, including validamycin and succinate dehydrogenase inhibitors (SDHIs), such as flutolanil and penthiopyrad [21,22]. For cabbage bottom rot caused by *R. solani*, a broader spectrum of fungicide classes is registered in Japan. These include QoIs and SDHIs, which target distinct sites of mitochondrial respiration, as well as the dicarboximide iprodione, the pyridinamine-type oxidative phosphorylation uncoupler fluazinam, and validamycin. In addition, soil disinfestation with dazomet is practiced to manage cabbage damping-off caused by *R. solani*.

In 2021, the Japanese Ministry of Agriculture, Forestry and Fisheries (MAFF) launched the MIDORI Strategy for Sustainable Food Systems, which aims to reduce the risk-weighted use of chemical pesticides by 10% by 2030 and by 50% by 2050 through the dissemination of integrated pest management and newly developed alternatives. Since the development and registration of new pesticides generally require a long period of time, alternative and complementary disease management approaches that can be implemented relatively rapidly are urgently needed.

Lecithin is a complex mixture of phospholipids whose major components include phosphatidylcholine (PC), phosphatidylethanolamine (PE), phosphatidylinositol (PI), and phosphatidic acid (PA), together with other minor phospholipid species [23]. Because phospholipids possess both hydrophilic and hydrophobic domains, lecithin exhibits amphiphilic properties and can function as an emulsifier. Accordingly, lecithin has long been used as a natural emulsifier in a wide range of applications, including food products, cosmetics, and pharmaceuticals [23,24,25]. Commercial lecithin is produced mainly from plant oils, particularly soybean oil, as well as from egg yolk and other biological sources.

Previous studies at RIKEN demonstrated that soybean lecithin suppresses disease development caused by the rice blast fungus and powdery mildew fungi [26,27,28]. In the rice blast fungus, soybean lecithin inhibited penetration by infection hyphae at 5 ppm and markedly suppressed sporulation at concentrations above 10 ppm, whereas its inhibitory effect on mycelial growth was weak even at 500 ppm; furthermore, appressorium formation was inhibited at concentrations above 500 ppm and completely suppressed at 1000 ppm [28]. Soybean lecithin has also been reported to affect the development of the cucumber powdery mildew fungus, *Sphaerotheca fuliginea* [27,29]. These findings suggest that soybean lecithin can interfere with specific infection-related developmental processes in plant-pathogenic fungi. A commercial formulation containing soybean lecithin, consisting of 30% soybean lecithin and 70% butyl carbitol as a solvent, was registered as a pesticide in Japan in the 1970s, although its registration later lapsed. Nevertheless, because soybean lecithin is a naturally derived material and is listed among substances exempted from pesticide residue standards in Japan, it holds potential as an environmentally low-impact agricultural material compatible with sustainable crop protection.

In light of these considerations, we evaluated the efficacy of foliar application of soybean lecithin against foliar diseases caused by different *R. solani* isolates in cabbage and rice, both of which are agriculturally important crops affected by this pathogen in Japan. In addition, we used *B. distachyon* as an experimental model, as in our previous studies. Lecithin treatment reduced disease symptoms in these plants, which coincided with reduced hyphal development on inoculated leaves. We further examined whether the disease-suppressive activity of lecithin against *R. solani* was primarily due to direct effects on fungal hyphae or to the induction of plant defense responses. Our results suggest that the suppressive effect is driven, at least in part, by direct inhibition of *R. solani* hyphae, although the possible involvement of plant responses cannot be excluded. Furthermore, we also found that high concentrations of lecithin can cause phytotoxicity, suggesting that the effective concentration range should be carefully optimized for each crop.

## 2. Materials and Methods

### 2.1. Plant Materials and Growth Conditions

Seeds of cabbage (*Brassica oleracea* cv. Seirin) were purchased from Sakata Seed Corporation (Yokohama, Japan), and seeds of cabbage cvs. Ayahikari and Yumeibuki were purchased from Takii & Co., Ltd. (Kyoto, Japan). Seeds of rice (*Oryza sativa* cv. Nipponbare) and *B. distachyon* accession Bd21 were obtained from the National Agriculture and Food Research Organization (NARO) Genebank and the RIKEN Center for Sustainable Resource Science (CSRS), Japan, respectively. Cabbage seeds were sown directly in soil in 5 cm square plastic pots with 25 cells (Nichiei-Sangyo, Sano, Japan) placed in 27 cm square half trays (Jiffy, Zwijndrecht, The Netherlands) and grown in a growth chamber at 23 °C under a 16 h light/8 h dark photoperiod (BiOTRON NKsystem LH-411PFD, Osaka, Japan). Rice and *B. distachyon* seeds were surface-sterilized with 1% sodium hypochlorite for 5 min and rinsed three times with distilled water for 1 min each. After incubation on moist filter paper in Petri dishes covered with aluminum foil at 4 °C for 3 days, the seeds were transferred to a growth chamber under the same conditions. Germinated seedlings were transplanted into soil and grown under the same conditions. The soil used was Sakata Super Mix A (Sakata Seed Corporation) for cabbage and *B. distachyon*, and Suito Engei Baido Danchi-version (Yanmar Agri, Okayama, Japan) for rice.

### 2.2. Fungal Isolates and Culture Conditions

*R. solani* AG-1 IA isolate C-325 (MAFF 305230), originally isolated from rice, was obtained from the NARO Genebank, Japan. *Rhizoctonia* sp. RhiCa-2 was isolated from diseased cabbage plants in a field located in Gunma Prefecture, Japan, as described below. The isolates were grown on potato dextrose agar (PDA; potato dextrose broth supplemented with Bacto agar; BD Difco, Franklin Lakes, NJ, USA) at 23 °C. The infectivity of *R. solani* AG-1 IA isolate C-325 to plantlets and detached leaves of *B. distachyon* has been demonstrated in our previous studies [9,12,13,15].

### 2.3. Lecithin Treatment and Leaf Inoculation Assay

Leaves were detached from plants and placed on moist filter paper in Petri dishes (9 cm diameter). For cabbage, 20-day-old plants were used. For *B. distachyon* and rice, 3-week-old plants were generally used. Soybean lecithin (SLP-white, Tsuji Oil Mills Co., Ltd., Matsusaka, Japan) or egg lecithin (124-05031, Fujifilm Wako Chemicals, Osaka, Japan) was dispersed in water at 0.2, 0.5, 1.0, or 5.0% (*w*/*v*) with 0.1% (*v*/*v*) spreader (Approach BI; Maruwa Biochemical, Tokyo, Japan) and sprayed onto the detached leaves. The sprayed leaves were kept in a fume hood until dry. Mycelial plugs (4 mm diameter) were prepared from the edge of actively growing colonies of *R. solani* isolates using a biopsy punch (BP-40F, Kai Industries Co., Ltd., Tokyo, Japan) and placed at the center of each detached leaf. A PDA plug without mycelium was used as a control. The inoculated leaves were incubated in a growth chamber under high-humidity conditions for several days, and symptoms were observed daily. The leaves were photographed, and lesion area percentage was quantified using ImageJ software (v 1.54p) [30].

### 2.4. Identification of the Anastomosis Group of R. solani

Fungal hyphae of *Rhizoctonia* sp. RhiCa-2 and *R. solani* AG-1 IA and AG-4 HG-I+II isolate BO-3 (MAFF 305225) were collected from PDA medium and placed in tubes containing four zirconia beads (3 mm diameter). The hyphal samples were snap-frozen in liquid nitrogen and homogenized using a MicroSmash MS-100 homogenizer (TOMY SEIKO, Tokyo, Japan). Genomic DNA was extracted using the NucleoSpin Plant II kit (Takara Bio, Kusatsu, Japan), and DNA concentration and quality were assessed using a DS-11 spectrophotometer (DeNovix, Wilmington, DE, USA). PCR was performed using primers specific for each AG and subgroup according to Misawa (2019) [31], and amplification of products of the expected sizes was confirmed by agarose gel electrophoresis. The primers used were as follows: for AG-1 IA, 5′-CCTTAATTTGGCAGGAGGG-3′ and 5′-GACTATTAGAAGCGGTTCA-3′ (58 °C, 540 bp); for AG-1 IB, 5′-TGTAGCTGGCCTTTTAAC-3′ and 5′-GGACTATTAGAAGCGGTTCG-3′ (58 °C, 580 bp); for AG-1 IC, 5′-GAGTTGTTGCTGGCCTCTGG-3′ and 5′-CCAAGTCAATGGACTATTG-3′ (58 °C, 550 bp); for AG-2-1, 5′-CAAAGGCAATRGGTTATTGGAC-3′ and 5′-CCTGATTTGAGATCAGATCATAAAG-3′ (60 °C, 480 bp); for AG-2-2 IIIB, 5′-AGGCAGAGRCATGGATGGGAG-3′ and 5′-ACCTTGGCCAMCCTTTTTATC-3′ (62 °C, 500 bp); for AG-2-2 IV, 5′-AGGCAGAGACATGGATGGGAA-3′ and 5′-CTTGGCCACCCMTTTTTTAC-3′ (62 °C, 500 bp); for AG-2-2 LP, 5′-AGGCAGAGAAACATGGATGGGC-3′ and 5′-CCTCCAATACCAAAGTGAAACCAAATC-3′ (62 °C, 400 bp); for AG-2-3, 5′-GTAGCTGGCTCATCGTTCTT-3′ and 5′-CATTTCCCTTGGCCACCTTTG-3′ (62 °C, 400 bp); for AG-2-BI, 5′-GGGGAATTTATTTGTTGTTTTTTGTAATAG-3′ and 5′-CAATGGACTATTAGAAGCA-3′ (55 °C, 510 bp); for AG-3 PT, 5′-CTGAACGCCTCTAAGTCAGAA-3′ and 5′-CTTGATTAATGCAACTCCC-3′ (50 °C, 480 bp); for AG-3 TM, 5′-CTGAACGCCTCTAAGTCAGAA-3′ and 5′-TCATTCTTGATCCACTAGTC-3′ (50 °C, 455 bp); for AG-4 HG-I, 5′-GGACCTACTCTCYTTGG-3′ and 5′-ACAGGGTGTCCTCAGCGA-3′ (55 °C, 420 bp); for AG-4 HG-II, 5′-GGACCTTCTACTCCCCCT-3′ and 5′-ACAGGGTGTCCTCAGCGA-3′ (55 °C, 420 bp); for AG-4 HG-III, 5′-GTTGTAGCTGGCATTTCC-3′ and 5′-CCACCCCTCCCAAACTCT-3′ (58 °C, 560 bp); for AG-5, 5′-GGTTGTAGCTGGCTCATGAA-3′ and 5′-TGATACTCAAACAGGCATGC-3′ (55–58 °C, 350 bp); and for AG-6, 5′-CCCTCTGTCTACTCAATCCA-3′ and 5′-TGATACTCAAACAGGCATGC-3′ (55–58 °C, 230 bp).

### 2.5. RNA Extraction and Gene Expression Analysis

Detached *B. distachyon* leaves were sprayed with 0.2, 0.5, 1.0, or 5.0% lecithin and sampled after 24 h of treatment in tubes containing four zirconia beads (3 mm diameter), followed by immediate freezing in liquid nitrogen. The collected leaves were homogenized, and total RNA was extracted using the ISOSPIN Plant RNA kit (NIPPON GENE, Tokyo, Japan). RNA quantity and quality were assessed using a spectrophotometer. cDNA was synthesized using the PrimeScript RT Reagent Kit with gDNA Eraser (Takara Bio). Quantitative real-time PCR was performed with Taq Pro Universal SYBR qPCR Master Mix (Vazyme, Nanjing, China) on a LightCycler 96 Real-Time PCR System (Roche, Basel, Switzerland). The primers used were as follows: for *BdWRKY38* (*Bradi2g30695*), 5′-GGACACCTTCAGGGTGACAT-3′ and 5′-TTGTCGTCGTGGTAGGAGTG-3′ [12,14]. Gene expression levels were normalized to *BdUbi4* (*Bradi3g04730*), using primers 5′-TGACACCATCGACAACGTGA-3′ and 5′-GAGGGTGGACTCCTTCTGGA-3′ [32]. All experiments were performed with three biological replicates.

### 2.6. Hyphal Staining and Microscopic Analysis

Inoculated leaves of cabbage, *B. distachyon*, and rice were collected at 4, 3, and 8 days post-inoculation (dpi), respectively, and excised sections were placed in 2 mL tubes. The leaves were fixed using a graded ethanol series and incubated at 4 °C until chlorophyll was removed. They were then stained overnight with 0.1% (*w*/*v*) lactophenol trypan blue, and excess stain was removed by incubating the samples in a chloral hydrate solution (2.5 g/mL) until the tissue became fully transparent. The stained leaf samples were observed using a stereo microscope (ZEISS Stemi 305, Oberkochen, Germany).

### 2.7. Evaluation of the Effect of Lecithin on R. solani Growth

Cellophane membranes (9 cm diameter) were soaked in water and autoclaved. The membranes were then placed on 2.0% water agar (BA-10, INA, Ina, Japan) in Petri dishes. Lecithin solutions at concentrations of 0, 0.2, 0.5, 1.0, and 5.0%, each containing 0.1% (*v*/*v*) spreader (Approach BI), were evenly sprayed onto each cellophane membrane and briefly dried in a clean bench. Mycelial plugs were prepared from PDA medium colonized by *R. solani* AG-1 IA (MAFF 305230) using a biopsy punch (BP-40F, Kai Industries Co., Ltd.) and placed at the center of the cellophane membranes. The colony diameter was measured daily.

## 3. Results

### 3.1. Identification of the Causal Agent of Cabbage Bottom Rot in Gunma Prefecture, Japan

Seedling rot and bottom rot occurred in cabbage plants in Tsumagoi Village, Gunma Prefecture, Japan, and a fungal isolate designated RhiCa-2 was obtained from diseased cabbage tissues in 2017. Based on microscopic observation of the hyphae, the isolate was presumed to belong to *Rhizoctonia* sp. When the hyphae were observed by confocal microscopy after staining with Hoechst 33342 and SR2200, multiple nuclei were detected in each cell (Figure S1A), similar to *R. solani* AG-1 IA, which was used as a control. Thus, we concluded that the causal agent was a multinucleate *Rhizoctonia* isolate, rather than a binucleate *Rhizoctonia* isolate.

To further identify this causal agent, genomic DNA was extracted and analyzed by PCR using AG-specific primers covering AG-1 to AG-6 [31]. Amplification of a PCR product of the expected size was detected in RhiCa-2 only when primers specific for AG-1 IB were used (Figure S1B). *R. solani* AG-1 IA, AG-2-1 N1, and AG-4 HG-I+II were used as controls, and each was detected using its corresponding primer set.

**Figure 1:**
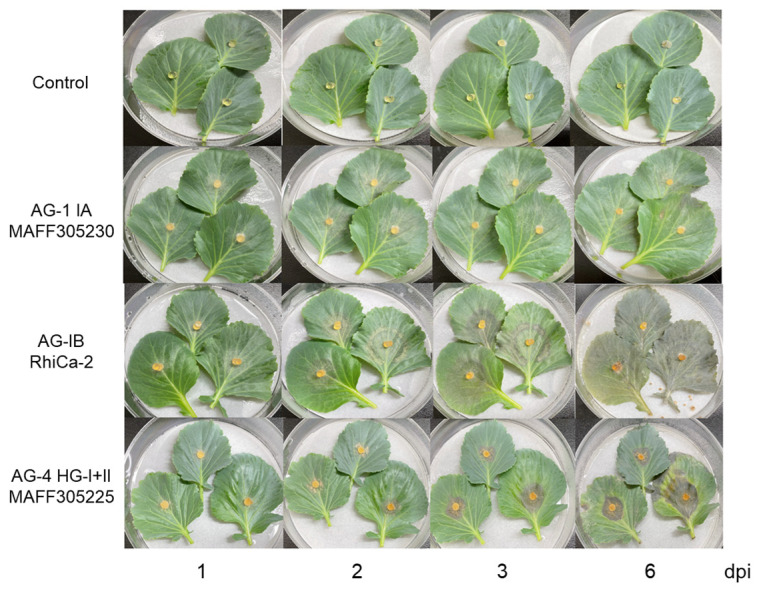
Evaluation of the virulence of *Rhizoctonia* sp. RhiCa-2 on cabbage leaves. Mycelial plugs of *R. solani* AG-1 IA, RhiCa-2, or AG-4 HG-I+II were inoculated onto detached leaves of the cabbage cultivar Seirin placed in Petri dishes lined with moist filter paper. Photographs were taken at 1, 2, 3, and 6 dpi.

When RhiCa-2 was inoculated onto detached leaves of three cabbage cultivars (Seirin, Ayahikari, and Yumeibuki), necrotic symptoms spread over almost the entire leaf area within 6 days (Figure 1; Figure S2A,B). Among the control isolates, AG-1 IA showed little virulence, whereas AG-4 HG-I+II caused necrosis around the inoculated mycelial plugs (Figure 1; Figure S2A,B). The expansion rate of the necrotic area caused by RhiCa-2 was higher than that caused by AG-4 HG-I+II. In addition, symptoms caused by RhiCa-2 spread uniformly, whereas those caused by AG-4 HG-I+II appeared patchy. The same fungal isolate was recovered from symptomatic tissues and reproducibly caused disease in cabbage. Based on these results, we concluded that *Rhizoctonia* sp. RhiCa-2, isolated from diseased cabbage in Gunma Prefecture, was *R. solani* AG-1 IB.

### 3.2. Effect of Lecithin Spraying on Disease Progression in Leaves Caused by R. solani

**Figure 2:**
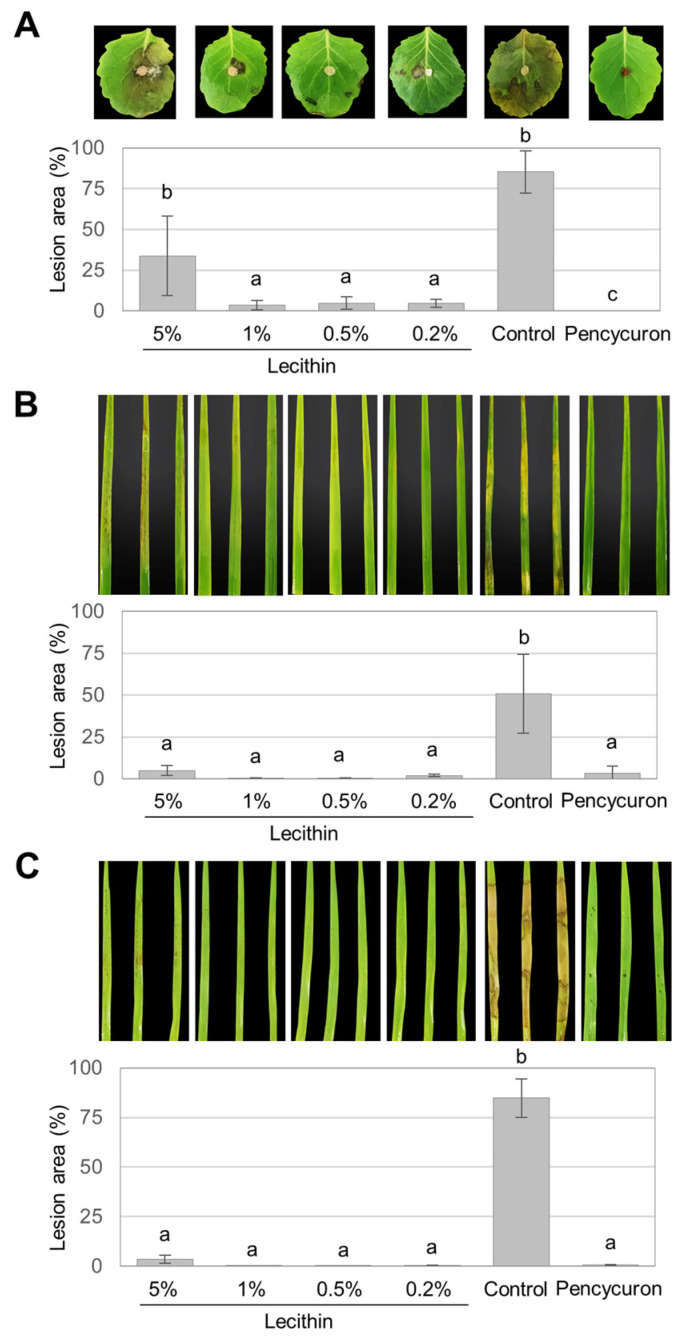
Evaluation of foliar treatment of soybean lecithin for suppressing disease caused by *Rhizoctonia solani* in plants. (**A**–**C**) Different concentrations of lecithin (0.2, 0.5, 1.0, and 5.0%) were sprayed onto detached leaves of cabbage (**A**), rice (**B**), and *Brachypodium distachyon* (**C**), and photographs were taken at 6 days post-inoculation. Representative images are shown for each concentration, and the corresponding lesion areas are presented as bar graphs below. A spreader-only solution and the fungicide pencycuron were used as negative and positive controls, respectively. Data are presented as mean ± SD (*n* = 3). Different letters indicate statistically significant differences, as assessed by one-way ANOVA followed by Tukey’s HSD post hoc test (*p* < 0.01).

Because lecithin has been used as an agrochemical for the control of powdery mildew, we hypothesized that it could suppress foliar symptoms caused by *R. solani* in plants. We first tested its effect on cabbage. Soybean lecithin was sprayed onto cabbage leaves at concentrations of 0.2, 0.5, 1.0, and 5.0%, followed by inoculation with *R. solani* AG-1 IB isolate RhiCa-2. Lecithin at 0.2–1.0% exhibited disease-suppressive activity, with a significant reduction in lesion area (Figure 2A). At 5.0%, lecithin also reduced the lesion area, but the effect appeared weaker than that observed at lower concentrations.

We further tested the activity of lecithin against *R. solani* diseases in monocotyledonous plants, namely rice and *B. distachyon*. Lecithin was sprayed onto detached leaves of rice and *B. distachyon* at the same concentration range, followed by inoculation with *R. solani* AG-1 IA. As shown in Figure 2B,C, 0.2, 0.5, and 1.0% lecithin suppressed symptoms in both plant species. These results indicate that lecithin can suppress foliar symptoms caused by *R. solani* in both dicotyledonous and monocotyledonous plants, with a similar effective concentration range among the plant species tested.

### 3.3. Mycelial Growth of R. solani on Lecithin-Treated Leaves

**Figure 3:**
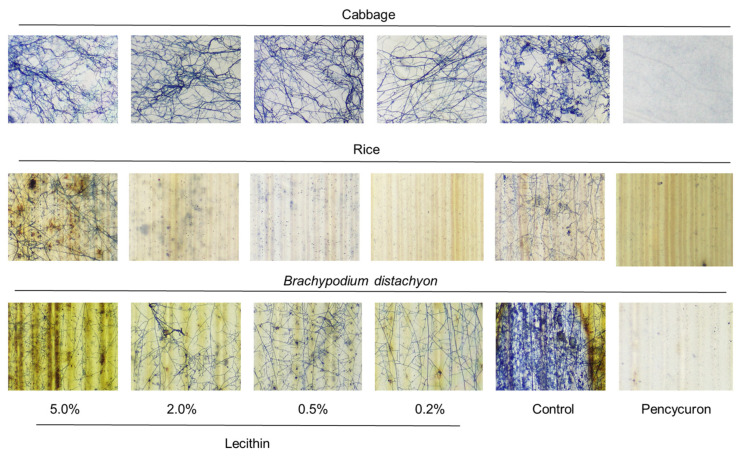
Detection of hyphae of *Rhizoctonia solani* isolates on plant leaves treated with different concentrations of soybean lecithin. Detached leaves of cabbage (***upper***), rice (***middle***), and *Brachypodium distachyon* (***lower***) were sprayed with 0.2, 0.5, 1.0, or 5.0% lecithin, and photographs were taken at 6 days post-inoculation with *R. solani* isolates. RhiCa-2 was used for cabbage, and AG-1 IA was used for rice and *B. distachyon*. Inoculated leaves were collected at 4, 8, and 3 dpi for cabbage, rice, and *B. distachyon*, respectively, fixed, stained with trypan blue, and observed using a stereo microscope.

To investigate the mechanism underlying lecithin-mediated disease suppression against *R. solani*, hyphal growth on lecithin-treated leaves was observed microscopically. Pathogen-inoculated leaves of cabbage, rice, and *B. distachyon* treated with different concentrations of lecithin were sampled, and fungal hyphae were stained with trypan blue. Whereas extensive hyphal spread was clearly observed in the control leaves, hyphal density decreased in the lecithin-treated leaves (Figure 3). In particular, numerous infection cushions were observed at the sampling time point in the control monocotyledonous plants, whereas the formation of such infection structures appeared to be inhibited by lecithin treatment.

### 3.4. Suppression of Hyphal Growth of R. solani on Cellophane by Lecithin

**Figure 4:**
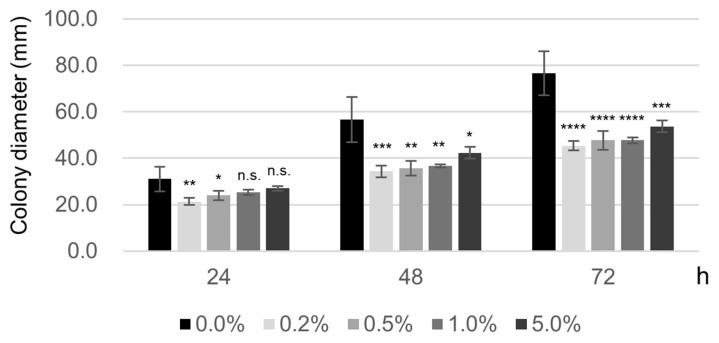
Direct effect of lecithin on hyphal elongation of *R. solani* AG-1 IA. Lecithin solutions at different concentrations (0.2, 0.5, 1.0, and 5.0%), each containing 0.1% (*v*/*v*) spreader, were sprayed onto a cellophane membrane placed on water agar. An inoculum of *R. solani* AG-1 IA was placed at the center of the membrane, and the diameter of the mycelial colony was measured daily. Water containing only the spreader was used as the control. Data are presented as mean ± SD (*n* = 3). Statistical significance was assessed by one-way ANOVA followed by Dunnett’s multiple comparison test against the 0% (untreated) control at each time point. Asterisks indicate significant differences from the 0% control: *, *p* < 0.05, **, *p* < 0.01, ***, *p* < 0.001, ****, *p* < 0.0001; n.s., not significant.

To further confirm the inhibitory effect of lecithin on fungal growth, we sprayed lecithin solutions onto cellophane sheets placed on water agar and then observed the hyphal elongation of *R. solani* AG-1 IA. Hyphal growth rate decreased significantly on the lecithin-treated cellophane (Figure 4). This reduction in growth rate was observed at all lecithin concentrations tested, with no obvious differences among them.

### 3.5. Phytotoxic Activity of Lecithin on B. distachyon

**Figure 5:**
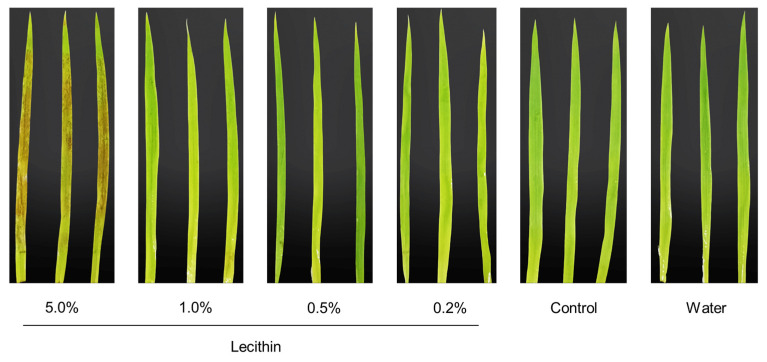
Phytotoxic activity of soybean lecithin on *Brachypodium distachyon*. Different concentrations of lecithin (0.2, 0.5, 1.0, and 5.0%) were sprayed onto detached leaves of *B. distachyon*, and photographs were taken at 6 days post-treatment. Water and a solution containing only the spreader were used as controls.

We examined the phytotoxic activity of lecithin in plants. Different concentrations of lecithin were sprayed onto detached *B. distachyon* leaves, which were then observed daily. At 6 days post-treatment, leaves treated with 5.0% lecithin showed browning symptoms (Figure 5). This result suggests that high concentrations of lecithin have a negative effect on plant viability. Thus, 5% lecithin is outside the appropriate concentration range for disease suppression. Although phytotoxic activity was characterized only in *B. distachyon* in this study, further characterization of its effects on rice, cabbage, and other plant species, as well as its detailed effects on plant tissues and the underlying mechanisms, will be needed in future studies.

### 3.6. Effect of Lecithin on Defense-Related Gene Expression in B. distachyon

We also examined whether lecithin treatment affects the expression of defense-related genes in plants using *B. distachyon*. Lecithin (0.2, 0.5, 1.0, and 5.0%) was sprayed onto *B. distachyon* leaves, and the expression of *BdWRKY38* was analyzed by reverse transcription-quantitative PCR (RT-qPCR) using cDNA prepared from RNA extracted from lecithin-treated leaf samples. The expression level of *BdWRKY38* was not significantly induced by lecithin treatment compared with the control, at least at the time point tested (Figure S3).

### 3.7. Disease-Suppressive Effect of Egg Lecithin Against R. solani

**Figure 6:**
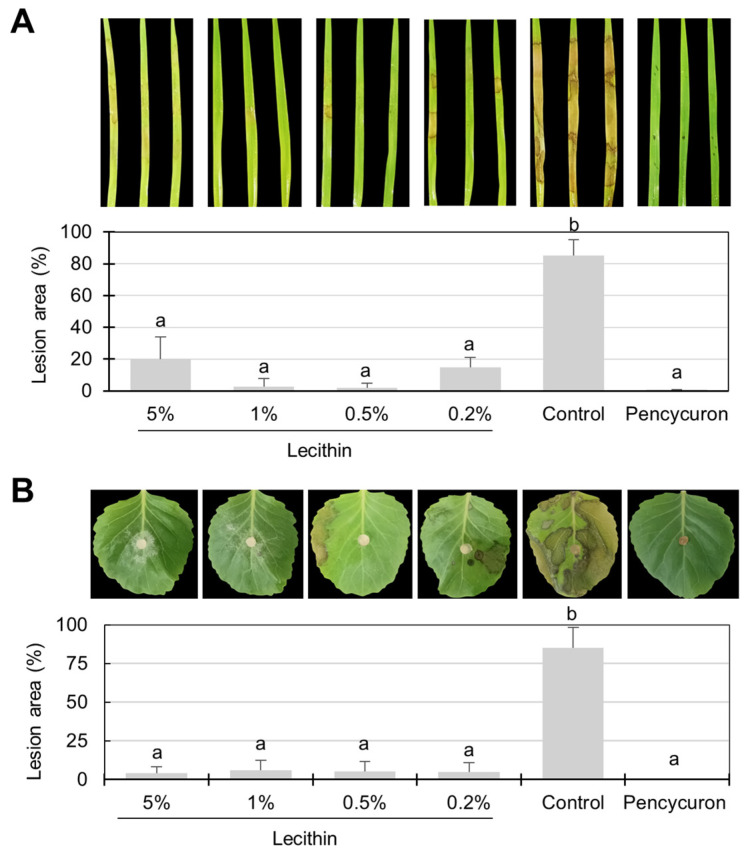
Evaluation of foliar treatment of egg lecithin for suppressing disease caused by *Rhizoctonia solani* in *Brachypodium distachyon* and cabbage. (**A**,**B**) Different concentrations of lecithin (0.2, 0.5, 1.0, and 5.0%) were sprayed onto detached leaves of *B. distachyon* (**A**) and cabbage (**B**), and photographs were taken at 6 days post-inoculation. Representative images are shown for each concentration, and the corresponding lesion areas are presented as bar graphs below. A spreader-only solution and the fungicide pencycuron were used as negative and positive controls, respectively. Data are presented as mean ± SD (*n* = 3). Different letters indicate statistically significant differences, as assessed by one-way ANOVA followed by Tukey’s HSD post hoc test (*p* < 0.01).

We also evaluated the disease-suppressive activity of egg yolk-derived lecithin on *B. distachyon* and cabbage. Detached leaves of *B. distachyon* and cabbage were sprayed with different concentrations of egg lecithin (0.2, 0.5, 1.0, and 5.0%), and symptoms caused by *R. solani* AG-1 IA and the AG-1 IB isolate RhiCa-2, respectively, were assessed. Egg lecithin also effectively suppressed symptoms in both plant species (Figure 6), with an efficacy comparable to that of soybean lecithin under the tested conditions. These results suggest that egg lecithin possesses potential as an alternative material for controlling Rhizoctonia diseases.

## 4. Discussion

This study revealed that *Rhizoctonia* sp. RhiCa-2 isolate, obtained from lesions on cabbage plants in Tsumagoi Village, Gunma Prefecture, was *R. solani* AG-1 IB. Damping-off caused by *Rhizoctonia*, *Pythium*, and *Fusarium* spp. is usually managed simply by removing diseased plants from the field unless severe damage occurs; therefore, the causal pathogens are often not precisely identified in practice. In cabbage, however, *Rhizoctonia* disease occurs not only as damping-off but also as bottom rot. Thus, the fungal population density in the field may increase, potentially leading to severe disease outbreaks under favorable conditions.

Fourteen *Rhizoctonia* isolates obtained from lesions on cabbage have been registered in the MAFF Genebank. The MAFF 245727 isolate, obtained in Hokkaido in 2015, was identified as AG-1 IC [33]. In addition, four isolates obtained in Iwate Prefecture in 2021 were identified as AG-2-1 and AG-2-2 IV [34]. Other isolates include AG-1 IA and AG-4 HG-I. Among these previously reported *Rhizoctonia* isolates infecting cabbage, two belong to AG-1 IB [MAFF 242306 (isolate CL1) and MAFF 242988 (isolate 09SKC-3)], both of which were isolated from different fields in Hokkaido. *R. solani* AG-1 IA isolates collected from cabbage (MAFF 237424), rice (isolate Cs-Gi), and timothy [MAFF 243451 (isolate MT-2)] showed pathogenicity when inoculated onto cabbage in the field [35,36]. To our knowledge, RhiCa-2 represents the first AG-1 IB isolate from cabbage identified in Honshu, the largest island of Japan.

The AGs of *R. solani* isolated from cabbage are notably diverse. This is consistent with previous studies suggesting that there is no strict correlation between pathogenicity and AG classification. One possible explanation is that, among fungal strains adapted to local environmental conditions (such as soil composition, pH, and soil temperature), strains retaining pathogenicity toward cabbage become established as causal agents in each region, and this trait may not be strictly linked to AG classification.

Given the diversity of AGs and pathogenicity mechanisms in *R. solani*, control strategies effective across different isolates and host plants would be highly valuable. In this study, we evaluated the efficacy of soybean lecithin in controlling *Rhizoctonia* disease as a potential management strategy that could contribute to achieving the SDGs and to the Strategy for Sustainable Food Systems, MIDORI. Previous studies have reported the disease-control effects of lecithin against the rice blast fungus and powdery mildew fungi [26,27,28,37,38,39]. More recently, the crop protection activity of lecithin against grapevine downy mildew was reported [40]. However, its effects against *Rhizoctonia* diseases have not been extensively investigated. Our results demonstrated that lecithin exhibited control efficacy against foliar infection by *R. solani* in cabbage, rice, and *B. distachyon* under laboratory conditions.

We previously reported that pretreatment with salicylic acid confers disease control against AG-1 IA infection in rice and *B. distachyon* [12,13]. In contrast, we also showed that salicylic acid pretreatment does not induce resistance against any of the AGs tested, including AG-1 IA, in Arabidopsis [8], or against AG-4 HG-I+II in rice or *B. distachyon* [9]. These findings suggest that *R. solani* comprises diverse anastomosis groups and that its pathogenicity mechanisms are similarly diverse. Although the infection mechanism of *R. solani* AG-1 IB isolate RhiCa-2 in cabbage, as well as the effects of plant hormones on resistance induction against this isolate, remain unclear, a disease-control effect of lecithin was commonly observed against all isolates tested in this study. These results suggest that lecithin may have broad applicability against *R. solani* isolates, although further validation using additional isolates is required.

Furthermore, because induction of the defense-related gene *BdWRKY38* was not detected after lecithin treatment, salicylic acid-dependent defense responses may not be induced by lecithin in *B. distachyon*, at least under the conditions tested. Thus, the suppressive effect of lecithin on lesion formation caused by *Rhizoctonia* is likely attributable, at least in part, to a direct effect on fungal hyphae. Consistent with this interpretation, hyphal density was lower on lecithin-treated leaves than on control leaves. Inhibition of hyphal elongation was also observed when lecithin was applied to cellophane placed on water agar. Because conditions such as moisture availability differ between the leaf surface and cellophane on water agar, this assay does not fully reproduce the action of lecithin on the leaf surface. Nevertheless, these results support the possibility that lecithin directly affects *Rhizoctonia* hyphae.

Given its amphiphilic properties, lecithin may disrupt biological cell membranes. This possibility could be examined in greater detail through follow-up experiments involving electron microscopic observation of lecithin-treated hyphae. Indeed, previous studies on powdery mildew fungi have shown shrunken hyphal tips after lecithin treatment at 2000 ppm (0.2%) [27]. In addition, 0.05% lecithin (500 ppm) induced morphological abnormalities in conidia and germ tubes of *P. oryzae* [38]. These findings support the possibility that lecithin exerts a similar negative effect on *Rhizoctonia* hyphae. Interestingly, appressorial penetration by *P. oryzae* was almost completely prevented by lecithin even at 5 ppm [28,38].

In our study, infection cushion formation was observed in rice and *B. distachyon*, but not in cabbage. Specific infection-related structures of *R. solani*, such as infection cushions, may differ in their sensitivity to lecithin, although this possibility should be examined more directly. Therefore, for practical application, it would be useful to determine the optimal application concentration of lecithin for each crop.

Although the present results support a direct inhibitory effect of lecithin on fungal hyphae, the possibility that lecithin also induces other defense-related activities in plants cannot be excluded. Lecithin has been reported to induce fengycin lipopeptide production by beneficial bacteria, thereby contributing to the suppression of cucumber mosaic virus [41]. Lecithin may also exert indirect effects on plants through environmental microorganisms. Additionally, our experiments revealed phytotoxicity caused by lecithin at high concentrations. Stress conditions induced by lecithin treatment may trigger defense-related activities other than the salicylic-acid-dependent response examined, thereby contributing to protective activity against a certain range of pathogens.

Taken together, our findings support the potential applicability of lecithin for controlling *Rhizoctonia* diseases in multiple host plants. However, the precise mode of action of lecithin, including its direct effects on fungal hyphae, possible indirect effects on plant defense responses, and phytotoxic activity at high concentrations, remains to be clarified in future studies.

## Data Availability

All data are available in the manuscript.

## Supplementary Materials

The following supporting information can be downloaded at: https://www.mdpi.com/article/10.3390/life16060998/s1, Figure S1: Identification of fungal species of RhiCa-2 and its virulence evaluation on cabbage. (**A**) Fungal hyphae of *Rhizoctonia* sp. RhiCa-2 and *Rhizoctonia solani* AG-1 IA (MAFF305230) were stained with Hoechst 33342 and SR2200 (SCRI Renaissance Stain 2200) with or without propidium iodide and observed with confocal fluorescent microscopy. Multiple nuclei were observed in both fungal isolates. (**B**) Gel electrophoresis of PCR products amplified using primers that specifically detect AGs from AG-1 to AG-6. In addition to RhiCa-2 (lane 1), AG-1 IA (lane 2), AG-2-1 N1 (lane 3), and AG-4 HG-I+II (lane 4) were used as sources of template genomic DNA to confirm the validity of the experiment; Figure S2: Evaluation of virulence of *Rhizoctonia* sp. RhiCa-2 on cabbage cultivars. (**A**,**B**) Mycelial plugs of AG-1 IA, RhiCa-2, or AG-4 HG-I+II were inoculated to detached leaves of cabbage cultivars Ayahikari (**A**) or Yumeibuki (**B**), and photographs were taken at 1, 2, 3, and 6 dpi; Figure S3: Evaluation of induction of defense-related gene induction by lecithin treatment in *Brachypodium distachyon*. *B. distachyon* leaves were treated with lecithin (0.2, 0.5, 1.0 and 5.0%), and *BdWRKY38* expression was analyzed by RT-qPCR using *BdUbi4* as an internal control. *BdWRKY38* was used as a marker for salicylic acid-responsive defense signaling. Lecithin treatment did not significantly upregulate *BdWRKY38* expression was at any concentration tested. Salicylic acid was used as a positive control. Different letters indicate statistically significant differences, as assessed by one-way ANOVA followed by Tukey’s HSD post hoc test (*p* < 0.01).
