# Supplementary material for: Disease-Suppressive Activity of Lecithin Against Foliar Infection by Rhizoctonia solani Isolates in Cabbage, Rice, and Brachypodium distachyon
Source: Life (Basel). 2026 Jun 13;16(6):998. doi: 10.3390/life16060998 (PMC13301948; doi:10.3390/life16060998)
Supplement: Supplementary file 1 [file life-16-00998-s001.zip › life-4367483-supplementary.pdf]

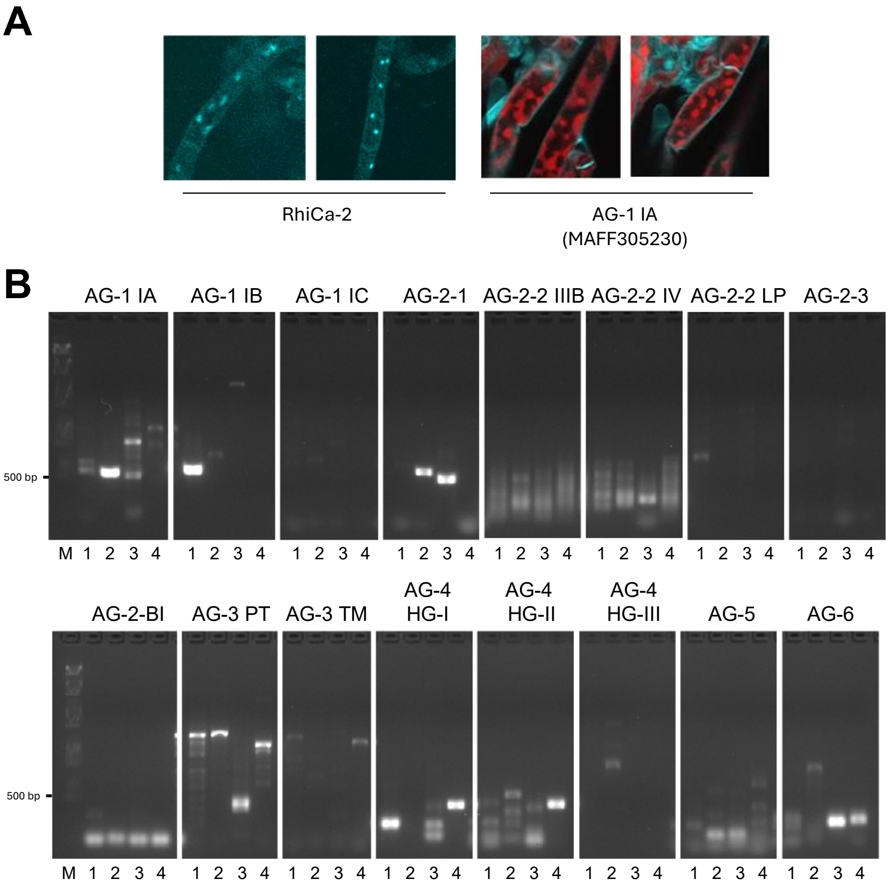


**Figure S1.** Identification of fungal species of RhiCa-2 and its virulence evaluation on cabbage. (**A**) Fungal hyphae of *Rhizoctonia* sp. RhiCa-2 and *Rhizoctonia solani* AG-1 IA (MAFF305230) were stained with Hoechst 33342 and SR2200 (SCRI Renaissance Stain 2200) with or without propidium iodide and observed with confocal fluorescent microscopy. Multiple nuclei were observed in both fungal isolates. (**B**) Gel electrophoresis of PCR products amplified using primers that specifically detect AGs from AG-1 to AG-6. In addition to RhiCa-2 (lane 1), AG-1 IA (lane 2), AG-2-1 N1 (lane 3), and AG-4 HG-I+II (lane 4) were used as sources of template genomic DNA to confirm the validity of the experiment.

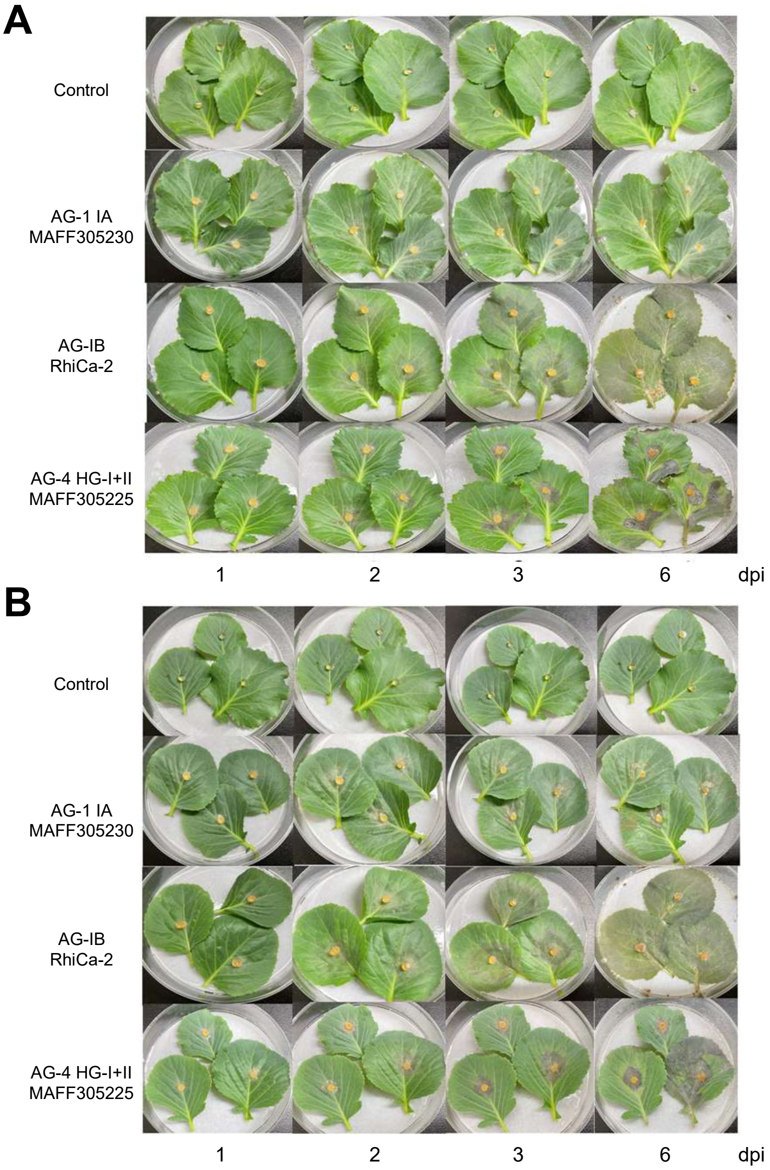


**Figure S2.** Evaluation of virulence of Rhizoctonia sp. RhiCa-2 on cabbage cultivars. (**A,B**) Mycelial plugs of AG-1 IA, RhiCa-2, or AG-4 HG-I+II were inoculated to detached leaves of cabbage cultivars Ayahikari (**A**) or Yumeibuki (**B**), and photographs were taken at 1, 2, 3, and 6 dpi.

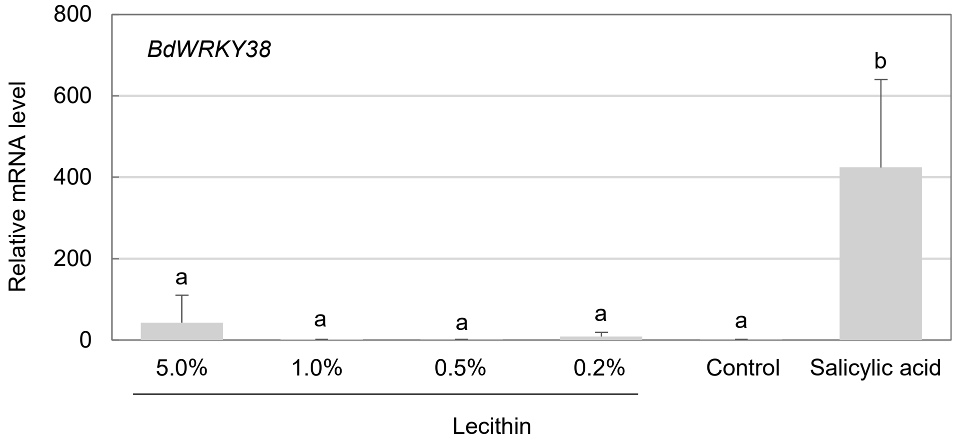


**Figure S3.** Evaluation of induction of defense-related gene induction by lecithin treatment in *Brachypodium distachyon*. *B. distachyon* leaves were treated with lecithin (0.2, 0.5, 1.0 and 5.0%), and *BdWRKY38* expression was analyzed by RT-qPCR using *BdUbi4* as an internal control. *BdWRKY38* was used as a marker for salicylic acid-responsive defense signaling. Lecithin treatment did not significantly upregulate *BdWRKY38* expression was at any concentration tested. Salicylic acid was used as a positive control. Different letters indicate statistically significant differences, as assessed by one-way ANOVA followed by Tukey's HSD post hoc test ($p < 0.01$).
